# High risk fertility behaviour and health facility delivery in West Africa

**DOI:** 10.1186/s12884-023-06107-1

**Published:** 2023-12-07

**Authors:** Eugene Budu, Bright Opoku Ahinkorah, Joshua Okyere, Abdul-Aziz Seidu, Richard Gyan Aboagye, Sanni Yaya

**Affiliations:** 1 Korle Bu Teaching Hospital, P.O. Box 77, Accra, Ghana; 2 REMS Consult Limited, Sekondi-Takoradi, Western Region Ghana; 3 School of Clinical Medicine, University of New South Wales, Sydney, Australia; 4 Department of Population and Health, University of Cape Coast, Cape Coast, Ghana; 5 Department of Nursing, College of Health Sciences, Kwame Nkrumah University of Science and Technology, Kumasi, Ghana; 6 Centre for Gender and Advocacy, Takoradi Technical University, Takoradi, Ghana; 7 College of Public Health, Medical and Veterinary Sciences, James Cook University, Townsville, Australia; 8 Department of Family and Community Health, Fred N. Binka School of Public Health, University of Health and Allied Sciences, Hohoe, Ghana; 9 School of International Development and Global Studies, University of Ottawa, Ottawa, Canada; 10 The George Institute for Global Health, Imperial College London, London, UK

**Keywords:** Health risk, Fertility behaviour, Health facility, Delivery, West Africa

## Abstract

**Background:**

Evidence suggests that women who give birth in a health facility have lower odds of experiencing pregnancy complications and significantly reduced risk of death from pregnancy-related causes compared to women who deliver at home. Establishing the association between high-risk fertility behaviour (HRFB) and health facility delivery is imperative to inform intervention to help reduce maternal mortality. This study examined the association between HRFB and health facility delivery in West Africa.

**Methods:**

Data for the study were extracted from the most recent Demographic and Health Surveys of twelve countries in West Africa conducted from 2010 to 2020. A total of 69,479 women of reproductive age (15–49 years) were included in the study. Place of delivery was the outcome variable in this study. Three parameters were used as indicators of HRFB based on previous studies. These were age at first birth, short birth interval, and high parity. Multivariable binary logistic regression analysis was performed to examine the association between HRFB and place of delivery and the results were presented using crude odds ratio (cOR) and adjusted odds ratio (aOR), with their respective 95% confidence interval (CI).

**Results:**

More than half (67.64%) of the women delivered in a health facility. Women who had their first birth after 34 years (aOR = 0.52; 95% CI = 0.46–0.59), those with short birth interval (aOR = 0.91; 95% CI = 0.87–0.96), and those with high parity (aOR = 0.58; 95% CI = 0.55–0.60) were less likely to deliver in a health compared to those whose age at first delivery was 18-34 years, those without short birth interval, and those with no history of high parity, respectively. The odds of health facility delivery was higher among women whose first birth occurred at an age less than 18 years compared to those whose age at first birth was 18-34 years (aOR = 1.17; 95% CI = 1.07–1.28).

**Conclusion:**

HRFB significantly predicts women's likelihood of delivering in a health facility in West Africa. Older age at first birth, shorter birth interval, and high parity lowered women’s likelihood of delivering in a health facility. To promote health facility delivery among women in West Africa, it is imperative for policies and interventions on health facility delivery to target at risk sub-populations (i.e., multiparous women, those with shorter birth intervals and women whose first birth occurs at older maternal age). Contraceptive use and awareness creation on the importance of birth spacing should be encouraged among women of reproductive age in West Africa.

## Background

Worldwide, maternal mortality remains a critical public health concern. The World Health Organization (WHO) report indicates that in 2017, nearly 810 women died from pregnancy-related causes every day [1]. In the same report, it was shown that more than two-thirds of maternal mortality (94%) occurred in low-and middle-income countries (LMICs) [1]. Sub-Saharan Africa (SSA) alone accounted for 66% of the global maternal mortality in 2017 [2]. The Sustainable Development Goal (SDG) target 3.1 indicates that countries need to prioritise maternal health in order to achieve a reduction in maternal mortality to 70 per 100,000 live births by 2030 [3]. In addition, the WHO’s supplementary national target states that by 2030, no country should have a maternal mortality ratio that is higher than 140 deaths per 100,000 live births [4]. To achieve these international goals on maternal health, it is imperative to improve access to antenatal care (ANC), obstetric referrals, skilled birth delivery, institutional birth deliveries, and postnatal care services (PNC).

Evidence suggest that women who deliver in a health facility have lower odds of experiencing pregnancy complications and significantly reduced risk of death from pregnancy-related causes compared to women who deliver at home [5–7]. Evidence from SSA [6], Indonesia [8], Uganda [9], Ghana [10], and India [11] have shown that the factors that predict women’s likelihood of delivering in a health facility include educational attainment, place of residence, employment status, frequency and timeliness of ANC attendance, women’s autonomy in making healthcare decisions, and exposure to media.

Beyond the aforementioned factors associated with health facility delivery [8–11], there are other factors such as high-risk fertility behaviour (HRFB) which may influence health facility deliveries. Fertility behaviour is concerned with maternal age, birth spacing, and birth order [12]. Consequently, HRFB refers to “too-early or too-late maternal age at delivery, shorter birth interval, and a higher number of live births” [13]. There are limited studies that have examined the individual components of HRFB and how they are associated with health facility delivery. For instance, a study conducted in Nigeria reported that women who had their first birth before age 20 had lower odds of delivering in a health facility [14]. Similarly, a multi-country study conducted in SSA reported that older age at first birth was associated with higher likelihood of health facility delivery [15]. Previous systematic reviews have also documented that higher parity is associated with reduced health facility deliveries [6, 16]. However, none of these cited studies examined all three components of HRFB in a single study thereby creating a gap in the current scholarship on the determinants of health facility deliveries. Hence, the need for further research. Examining the association between HRFB and health facility delivery is imperative to inform policy and intervention development to help reduce maternal mortality. This study examined the association between HRFB and health facility delivery in West Africa using nationally representative survey datasets.

## Methods

### Data source and study design

Data for the study were extracted from the most recent Demographic and Health Surveys (DHS) of twelve countries in West Africa conducted from 2010 to 2020 (Table 1). We pooled the data from the women’s recode files in each of the 12 countries. The DHS is a comparatively nationally representative survey conducted in over 90 low-and middle-income countries worldwide [17]. DHS employed a cross-sectional design. Respondents for the survey were recruited using a two-stage cluster sampling method. Detailed sampling technique has been highlighted in the literature [18]. Standardised structured questionnaires were used to collect data from the respondents on health indicators including place of delivery and fertility behaviour [17]. A total of 69,479 women in their reproductive age (15–49 years) were used in the study. The datasets used are freely available at https://dhsprogram.com/data/available-datasets.cfm. This paper was written with reference to the Strengthening the Reporting of Observational Studies in Epidemiology (STROBE) statement guidelines [19].

**Table 1:** Sample distribution per country

Country	Survey year	Weighted N	Weighted %
1. Benin	2017-18	7508	10.81
2. Burkina Faso	2010	8988	12.93
3. Côte d’Ivoire	2011-12	3392	4.88
4. Gambia	2019-2020	2454	3.53
5. Ghana	2014	2426	3.49
6. Guinea	2018	5746	8.27
7. Liberia	2019-2020	2780	4.00
8. Mali	2018	6955	10.01
9. Nigeria	2018	13,620	19.60
10. Senegal	2010-11	6268	9.02
11. Sierra Leone	2019	4935	7.10
12. Togo	2013-14	4407	6.34
**Total**	**2010–2020**	**69,479**	**100.00**

### Variables

#### Outcome variable

Place of delivery was the outcome variable in this study. This variable refers to the specific place where pregnant women gave birth to their recent child/children. This variable was derived from the question, “Where did you deliver in your last birth?” This was focused on live births to interviewed women in the 5 years preceding the surveys. Responses to this question were “respondent’s home”, “other home”, “government hospital”, “government health centre/clinic” “government health post/Community-Based Health Planning and Services (CHPS) compound” “other public”, “private hospital, clinic”, “maternity home” and “other”. For the purpose of this study, the variable was recoded into; “0” = “home delivery” which includes “respondent’s home” and “other home” and “1” = “facility delivery” which also comprised “government hospital”, “government health centre/clinic” “government health post/community-based health planning and services compounds” “other public”, “private hospital, clinic”, and “maternity home”. The ‘other’ category was dropped to ensure accuracy of the categorization into health facility and home delivery. The number of observations were less than 5%.

#### Explanatory variable

HRFB was the key explanatory variable in the study. Three parameters were used as indicators of HRFB based on previous studies [12, 13]. These were age at first birth less than 18 years or more than 34 years, short birth interval (less than 24 months interval between the current and preceding births), and high parity (women with more than 3 births).

### Covariates

The covariates considered in this study were selected based on their association with place of delivery from literature [5, 6, 10] and their availability in the DHS datasets. A total of 10 covariates were included in the study. These covariates were mother’s age, employment status, wealth index, religious affiliation, marital status, sex of household head, frequency of reading newspaper, frequency of listening to radio, frequency of watching television, and health insurance subscription.

### Statistical analyses

Data for the study was analysed using Stata version 16. First, a bar chart was used to show the proportion of women who delivered in a health facility across the 12 countries. Next, weighted frequencies and percentages for the explanatory variables and covariate were presented in Table 2. Later, we presented the bivariate results showing the distribution of health facility delivery across the explanatory variables and the covariates using chi-square test of independence (Table 2). Subsequently, two binary logistic regression models were used to examine the association between HRFB and place of delivery, controlling for the covariates. The first model, Model I, was a bivariate logistic regression analysis between each of the explanatory variables and place of delivery and the second model, Model II, was a multivariable logistic regression analysis, which had all the explanatory variables, covariates, and outcome variable in the same model (Table 3). The results were presented using crude odds ratio (cOR) and adjusted odds ratio (aOR), with their respective 95% confidence interval (CI). Statistical significance was set at *p* < 0.05 in the chi-square test and regression analysis. All the analyses were weighted and the survey command (svy) in Stata was used to adjust for the complex sampling structure of the data in the analyses. We restricted our analysis to complete cases, therefore, all missing values were dropped.

### Ethical consideration

In this study, ethical clearance was not sought due to the public availability of the DHS dataset. The datasets were obtained from the MEASURE DHS after registration and approval were given for its usage. All the ethical guidelines concerning the use of secondary datasets in the publication were strictly adhered to. Detailed information about the DHS data usage and ethical standards are available at http://goo.gl/ny8T6X.

## Results

### Prevalence of health facility delivery among women in West Africa

**Fig. 1:**
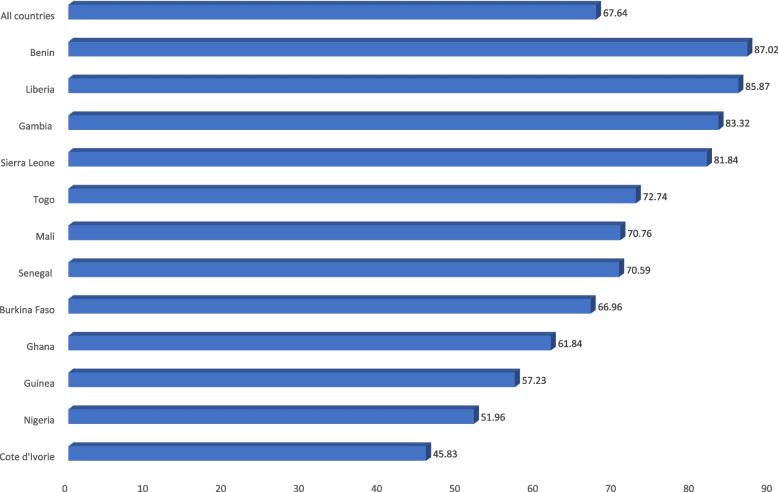
Prevalence of health facility delivery among women in West Africa

Figure 1 shows the prevalence of health facility delivery among women in west Africa. Overall, about 68 out of every 100 women gave birth in a health facility, with the highest prevalence in Benin (87.02%) and the lowest in Côte d’Ivoire (45.83%).

### High risk fertility behaviours, background characteristics and place of delivery

Table 2 shows the distribution of the proportion of health facility delivery across the studies variables. The results showed that 56.6% of the women aged more than 34 years delivered in a health facility whilst 69.0% of those aged below 18 years had health facility deliveries. Also, 63.8% of women with short birth interval gave birth in a health facility while 65.0% of those with high parity gave birth in a health facility. For the covariates, the proportion of health facility was lower among women from households with the poorest wealth index (54.6%), those belonging to the Traditional religion (64.9%), those married (65.9%), those in male-headed household (65.7%), those not exposed to newspaper (62.4%), radio (62.4%), television (63.5%) and those who had not subscribed to national health insurance (67.4%). With the exception of employment status, all the variables showed statistically significant differences with place of delivery.

**Table 2:** High risk fertility behaviours, background characteristics, and place of delivery

Variables	Weighted N	Weighted %	Health facility delivery	*p*-value
**Age at first birth**				< 0.001
Less than 18 years	2965	4.3	69.0	
18–34 years	65,323	94.0	67.8	
More than 34 years	1191	1.7	56.6	
**Short birth interval**				< 0.001
No (24 months and above)	60,219	86.7	68.2	
Yes (less than 24 months)	9260	13.3	63.8	
**High parity**				< 0.001
No	31,807	45.8	70.8	
Yes	37,672	54.2	65.0	
**Women's age**				< 0.001
15–24 years	16,409	23.6	64.9	
25 − 34 years	27,880	40.1	63.4	
35–49 years	25,190	36.3	74.1	
**Employment status**				0.467
Unemployed	13,424	19.3	68.4	
Employed	56,018	80.7	67.4	
**Wealth index**				< 0.001
Poorest	20,876	30.1	54.6	
Poorer	20,507	29.5	64.6	
Middle	16,497	23.7	74.5	
Richer	8849	12.7	84.7	
Richest	2750	4.0	93.2	
**Religious affiliation**				< 0.001
Christianity	63,436	91.3	67.4	
Islam	1608	2.3	81.0	
Traditionalist	3109	4.5	64.9	
No religion	1326	1.9	67.6	
**Marital status**				< 0.001
Never married	2650	3.8	77.4	
Married	57,772	83.1	65.9	
Cohabiting	5878	8.5	73.6	
Widowed	1413	2.0	81.8	
Divorced/separated	1794	2.9	79.7	
**Sex of household head**				< 0.001
Male	59,094	85.1	65.7	
Female	10,385	14.9	78.6	
**Frequency of reading newspaper**				< 0.001
Not at all	66,621	95.9	66.9	
Less than once a week	1922	2.7	81.9	
At least once a week	919	1.3	89.4	
Almost everyday	17	0.1	83.6	
**Frequency of reading newspaper**				< 0.001
Not at all	29,765	42.8	62.4	
Less than once a week	16,158	23.3	69.3	
At least once a week	22,579	32.5	72.8	
Almost everyday	977	1.4	81.4	
**Frequency of reading newspaper**				< 0.001
Not at all	47,401	68.2	63.5	
Less than once a week	10,827	15.6	72.3	
At least once a week	10,993	158	80.1	
Almost everyday	258	0.4	93.6	
**Health insurance subscription**				< 0.001
No	67,044	96.5	67.4	
Yes	2435	3.5	73.6	

### Association between high-risk fertility behaviour and place of delivery among women in West Africa

Table 3 shows the association between HRFB and place of delivery among women in West Africa. Women who had their first birth after 34 years (aOR = 0.52; 95% CI = 0.46–0.59), those with short birth interval (aOR = 0.91;95% CI = 0.87–0.96), and those with high parity (aOR = 0.58; 95% CI = 0.55–0.60) were less likely to deliver in a health facility compared to those whose age at first birth was 18-34 years, those without short birth interval, and those with no history of high parity, respectively. Women whose age at first birth was below 18 years were more likely to deliver in a health facility relative to those whose birth occurred during age 18 to 34 years (aOR = 1.17; 95% CI = 1.07–1.28). With the covariates, women aged 35–49 years, those in the richest wealth quintile, those belonging to Islamic religious sect, those in a female headed household, those who read newspaper at least once a week, those who listened to radio almost every day, and those who watched television almost every day were more likely to deliver in a health facility. However, married women and those who belonged to the African traditional religion had the lowest odds of health facility delivery (See Table 3).

**Table 3:** High risk fertility behaviours and place of delivery among women in West Africa

Variables	Model I [cOR (95% CI)]	Model II [aOR (95% CI)]
Age at first birth		
Less than 18 years	1.08 (1.00-1.17)	1.17^***^ (1.07–1.28)
18–34 years	Reference (1.0)	Reference (1.0)
More than 34 years	0.63^***^ (0.56–0.70)	0.52^***^ (0.46–0.59)
**Short birth interval**		
No (24 months and above)	Reference (1.0)	Reference (1.0)
Yes (less than 24 months)	0.83^***^ (0.80–0.87)	0.91^***^ (0.87–0.96)
**High Parity**		
No	Reference (1.0)	Reference (1.0)
Yes	0.78^***^ (0.75–0.80)	0.58^***^ (0.55–0.60)
**Women's age**		
15–24 years	Reference (1.0)	Reference (1.0)
25 − 34 years	0.93^***^ (0.89–0.97)	1.31^***^ (1.25–1.38)
35–49 years	1.54^***^ (1.48–1.61)	2.81^***^ (2.65–2.99)
**Wealth index**		
Poorest	Reference (1.0)	Reference (1.0)
Poorer	1.51^***^ (1.45–1.57)	1.43^***^ (1.38–1.49)
Middle	2.40^***^ (2.30–2.51)	2.16^***^ (2.06–2.26)
Richer	4.33^***^ (4.06–4.62)	3.71^***^ (3.47–3.98)
Richest	9.90^***^ (4.06–4.62)	7.00^***^ (5.99–8.20)
**Religious affiliation**		
Christianity	Reference (1.0)	Reference (1.0)
Islam	1.77^***^ (1.58–1.99)	1.82^***^ (1.62–2.05)
Traditionalist	0.80^***^ (0.74–0.86)	0.87^***^ (0.80–0.94)
No religion	0.90 (0.81–1.01)	0.96 (0.85–1.08)
**Marital status**		
Never married	Reference (1.0)	Reference (1.0)
Married	0.55^***^ (0.51–0.61)	0.61^***^ (0.56–0.68)
Cohabiting	0.82^***^ (0.74–0.91)	0.91 (0.81–1.02)
Widowed	1.24^**^ (1.06–1.44)	0.91 (0.77–1.08)
Divorced/separated	1.09 (0.95–1.26)	0.95 (0.82–1.10)
**Sex of household head**		
Male	Reference (1.0)	Reference (1.0)
Female	1.86^***^ (1.77–1.95)	1.46^***^ (1.38–1.54)
**Frequency of reading newspaper**		
Not at all	Reference (1.0)	Reference (1.0)
Less than once a week	2.23^***^ (1.98–2.50)	1.24^***^ (1.09–1.41)
At least once a week	3.60^***^ (2.96–4.38)	1.52^***^ (1.24–1.87)
Almost everyday	2.16 (0.62–7.58)	0.75 (0.23–2.42)
**Frequency of listening to radio**		
Not at all	Reference (1.0)	Reference (1.0)
Less than once a week	1.37^***^ (1.32–1.43)	1.19^***^ (1.14–1.29)
At least once a week	1.57^***^ (1.51–1.63)	1.29^***^ (1.23–1.34)
Almost everyday	2.25^***^ (1.51–1.63)	1.82^***^ (1.54–2.14)
**Frequency of watching television**		
Not at all	Reference (1.0)	Reference (1.0)
Less than once a week	1.51^***^ (1.44–1.58)	1.14^***^ (1.08–1.20)
At least once a week	2.23^***^ (2.12–2.35)	1.26^***^ (1.19–1.34)
Almost everyday	7.67^***^ (4.62–12.76)	2.82^***^ (1.68–4.72)
**Health insurance subscription**		
No	Reference (1.0)	Reference (1.0)
Yes	1.30^***^ (1.19–1.42)	0.99 (0.91–1.10)

^*^p < 0.05, ^**^p < 0.01, ^***^p < 0.001

## Discussion

This study examined the association between HRFB and health facility delivery. Our findings revealed that more than half of women (67.64%) delivered in a health facility. This finding is higher than the proportion of health facility delivery (50.6%) among women from seven countries in West and Central Africa [20]. Differences in the number of countries and survey years could have accounted for the higher proportion of health facility delivery in the current study relative to that of Olorunsaiye et al. [20]. Unlike the previous study that included only seven countries from West and Central Africa, we included 12 countries in West Africa with recent survey years. Nonetheless, the consensus between our study and Olorunsaiye et al.’s [20] study is that less than two-thirds of births in West Africa occur in health facilities. This is a disturbing finding because it poses a threat to West African countries’ capacity to achieve SDG targets 3.1 and 3.2, which aim at reducing maternal and neonatal mortalities, respectively [21]. The moderately low prevalence of health facility delivery could include the existence of disrespectful maternity care in some West African health facilities [22, 23], as well as inequalities in terms of accessibility to maternal healthcare services [24, 25].

Our finding indicates that women with high parity were less likely to have delivered in a health facility. Relatedly, studies conducted in Kenya [26] and Ghana [27, 28] have also found similar patterns of association between parity and health facility delivery where the likelihood of delivering in a health facility is significantly high among nulliparous women whilst the odds of delivering in a health facility reduced with additional parity. Probably, the findings that multiparous women are less likely to deliver in a health facility could be due to the perceived accumulated experience of childbirth, thereby reducing their pregnancy complication risk perception as compared to nulliparous women. Another perspective to this finding could be that unlike nulliparous women who may often be oblivious to the intricacies of childbirth, multiparous may have become overconfident as a result of previous childbirths [26]. Hence, reducing the value they place on delivering in a health facility.

Our study also revealed that older maternal age was associated with a significantly lower likelihood of delivering in a health facility. This result is consistent with Boah et al.’s [27] study that found the odds of delivering in a health facility to be significantly less among women of older maternal age. Our result is corroborated by another study conducted among Nepalese women that showed that younger women were more likely to deliver in a health facility compared to women of older maternal age [29]. We postulate that unlike younger women who are highly exposed to the media and other sources of health information, older women may lack or have insufficient knowledge about the need to have facility-based delivery. Older women are more likely to hold on to cultural beliefs of having their children delivered at home rather than at a health facility and this could have accounted for the observed finding in this study. Notwithstanding, our result contrasts with a study conducted in SSA that showed women of older maternal age having higher odds of delivering in health facilities [15].

Women with history of shorter birth intervals had lower odds of delivering in a health facility. The result is in alignment with Kawakatsu et al.’s [30] study which reported a lower likelihood of health facility delivery among women with history of short birth interval. A study conducted in Eritrea corroborates our findings by showing that women of wider birth intervals were 11 times more likely to deliver in a health facility compared to those with shorter birth intervals [31]. Our findings, thus, highlight a need for the governments, programme planners, and stakeholders in West African countries to strengthen existing interventions to advance the uptake of family planning to regulate their birth intervals.

Concerning the covariates, our study showed that exposure to the media was associated with higher likelihood of delivering in a health facility. This is consistent with a previous study [31]. A person’s beliefs and behaviours, particularly those regarding health issues, can be influenced by exposure to material on television, radio, and in print media [32]. This exposure can also boost knowledge and awareness of new concepts and social developments hence, explaining why women who are exposed to the media had higher odds to deliver in a health facility. Also, being in the richest wealth quintile was associated with higher likelihood to deliver in a health facility. A multi-country study involving seven West and Central African countries have also found a similar pattern of association between wealth status and the odds of delivering in a health facility [20]. This result may be explained from the point that women of higher wealth status tend to be empowered to take healthcare decisions such as the place of childbirth delivery. Moreover, women of higher wealth status have the economic resource to pay for health services that poorer women would not be able to afford. Women from traditionalist households were less likely to deliver in a health facility compared to those from Islamic households. Similar findings have been reported in Burkina Faso [33].

### Policy implications

Our findings underscore a need for West African countries to introduce policies and initiatives to reduce HRFB. Specifically, there is a need to deal with the unmet need for family planning in West Africa as that is a cost-effective means for women to optimise their birth intervals. Also, health education programmes have to be tailored to target multiparous women. Using the media (i.e., television and radio), multiparous women can be reached with health information and educational campaigns that advocate for health facility delivery.

### Strengths and limitations

The strength of this study lies in the use of the most recent nationally representative dataset from 12 West African countries. Also, the present study is the first of its kind to examine the association between and health facility delivery in the West African sub-region. However, some fundamental limitations must be considered in the interpretation of the findings. First, the use of a secondary dataset limited the number of variables that could be included in the study. For instance, the role of cultural beliefs and the normative system could not be explored. Also, the cross-sectional nature of the dataset does not allow us to establish causal association between HRFB and health facility delivery. Given the self-reported nature of the data, there is also the likelihood of self-reported bias.

## Conclusion

This study has shown that HRFB significantly predicts women likelihood of delivering in a health facility. Older age at first birth, shorter birth intervals, and higher parity are associated with lower likelihood of delivering in a health facility. To promote health facility delivery among women in West Africa, it is imperative for policies and interventions on health facility delivery to target at-risk sub-populations (i.e., multiparous women, those with shorter birth intervals, and women of older maternal age). Contraceptive use and awareness creation on the importance of birth spacing should be encouraged among women of reproductive age in West Africa.

## Data Availability

Data for this study were sourced from the MEASURE DHS and available here: https://dhsprogram.com/data/available-datasets.cfm. More details regarding DHS data and ethical standards are available at: http://goo.gl/ny8T6X.

## Abbreviations

ANC: Antenatal care
HRFB: High risk fertility behaviour
LMICs: Low-and-middle-income countries
PNC: Postnatal care
SDGs: Sustainable development goals
WHO: World health organization
